# Subsistence hunting impacts wildlife assemblages and functional ecology in tropical forests

**DOI:** 10.1038/s41598-025-87162-w

**Published:** 2025-01-24

**Authors:** Bradley Cain, Julia E. Fa, Rajan Amin, Jacqueline Morrison, Eva Avila Martin, Stephan M. Funk, Martin Jones, David P. Mallon, Robert Okale, Guillermo Ros Brull, Selvino R. de Kort

**Affiliations:** 1 Department of Natural Sciences, Faculty of Science and Engineering, Manchester Metropolitan University, Manchester, UK; 2 Center for International Forestry Research (CIFOR), Kota Bogor, Jawa Barat Indonesia; 3 Conservation Programmes, Zoological Society of London, London, UK; 4 North of England Zoological Society, Chester Zoo, Chester, UK; 5 Zerca y Lejos ONGD, Madrid, Spain; 6 Nature Heritage, Jersey, Channel Islands UK; 7 IUCN, Species Survival Commission, Gland, Switzerland

**Keywords:** Ecology, Conservation biology

## Abstract

Unsustainable wild meat hunting poses a significant threat to wildlife and tropical forest ecosystems. While high levels of extraction linked to commercial trade have received significant attention, the sustainability of subsistence hunting by Indigenous Peoples in Africa has been less studied. Understanding how changing lifestyles, particularly the sedentarisation of former hunter-gatherers, have affected the use of forest resources is crucial for wildlife conservation and livelihoods. The spatial management of hunting through the establishment of no-take zones, which act as sources for adjacent hunting areas, offers promise for the sustainability of Indigenous livelihoods. We conducted an extensive camera trap study in hunting areas subject to source-sink dynamics used by 10 sedentarised Baka communities. We compared species richness, occupancy, abundance, and community composition to a relatively non-hunted reference area in the adjacent Dja Faunal Reserve. Subsistence hunting by the Baka had a limited impact on species richness but significantly altered community composition and the abundance of carnivores, seed dispersers and granivores. These changes highlight that even the spatial management of hunting may have consequences for the sustainability of hunting systems and the functional ecology of tropical forests.

**Supplementary Information:**

The online version contains supplementary material available at 10.1038/s41598-025-87162-w.

## Introduction

Unsustainable hunting for food is a major driver of wildlife declines in the tropics^1^. The increased urban demand for wild meat and sophisticated hunting techniques has created ‘empty forests’ and ‘halos of defaunation’ in many areas^2–4^. However, many Indigenous and local communities still depend on subsistence hunting^5,6^. This creates a complex challenge when it comes to balancing human needs with the goals of biodiversity preservation and ecosystem protection^7,8^.

Although the rise of the wild meat trade in the tropics has received significant attention, the sustainability of hunting by Indigenous Peoples remains poorly understood. Sustainable hunting requires that wildlife populations are harvested at levels they can naturally replenish through reproduction. When hunting exceeds this threshold, sustainability depends on source-sink dynamics, where declining populations in overhunted areas are replenished by individuals from healthier populations in surrounding regions without changing the community composition^9,10^. This principle highlights the need for effective management of hunting areas to maintain the viability of target species while advancing broader conservation goals. Key strategies include establishing designated no-take zones, which serve as wildlife refuges and sources to sustain populations in adjacent hunting areas^11^.

Hunting pressure can disrupt trophic webs, resulting in the decline of large-bodied mammals and birds (especially large carnivores and seed dispersers) and an increase in granivores and mesopredators through ecological release^12,13^. This poses a significant threat to the regeneration and long-term persistence of tropical forests^14,15^. Assessing the sustainability of traditional subsistence hunting, characterised by minimal economic trade and a historical reliance on forest resources, is essential for ensuring the resilience of both local communities and ecosystems. Implementing spatial hunting management offers a promising framework to support the long-term well-being of these communities while safeguarding the forests they depend on^11,16^.

The impact of hunting by Indigenous Peoples can be measured by determining the differences in faunal composition in their hunting territories compared to a reference site where hunting is either absent or much less^17^. In this paper, we focus on Baka Pygmy communities that were sedentarised over the last half-century. These groups no longer conduct a traditional nomadic hunter-gatherer lifestyle but hunt regularly at relatively short distances from their villages^18^. Such a shift to central place foraging will likely increase hunting pressure on local areas, potentially leading to more pronounced defaunation effects and changes in wildlife assemblages. Our previous work in ten of these communities, on the Djoum-Mintom road (N9) south of the Dja Faunal Reserve (DFR) in the south-eastern Cameroon protected area, employed participatory mapping, GPS tracking, and community-based reporting to map hunting territories^18^. Community-based reporting schemes quantified offtake and techniques used by hunters within the hunting territories^19^. Here, we characterise the terrestrial larger-bodied mammal communities in the Baka hunting territories by employing an extensive camera trapping survey and comparing this with the results of a comparative camera trap survey in neighbouring DFR; an area where hunting pressure is minimal. We record mammal richness, diversity, guild structure, and relative abundance of taxa measured by trapping rates and occupancy of the two communities. Our research objectives include: (1) Evaluating the sustainability of wild meat extraction within Baka hunting territories which are subject to source-sink dynamics; (2) Investigating the influence of wild meat hunting on species richness; (3) Assessing whether centralised living patterns contribute to defaunation effects; (4) Exploring potential correlations between hunting preferences and species abundance and (5) Analysing the potential impacts of hunting offtake on functional diversity and shifts in wildlife communities.

## Materials and methods

### Study area

**Fig. 1:**
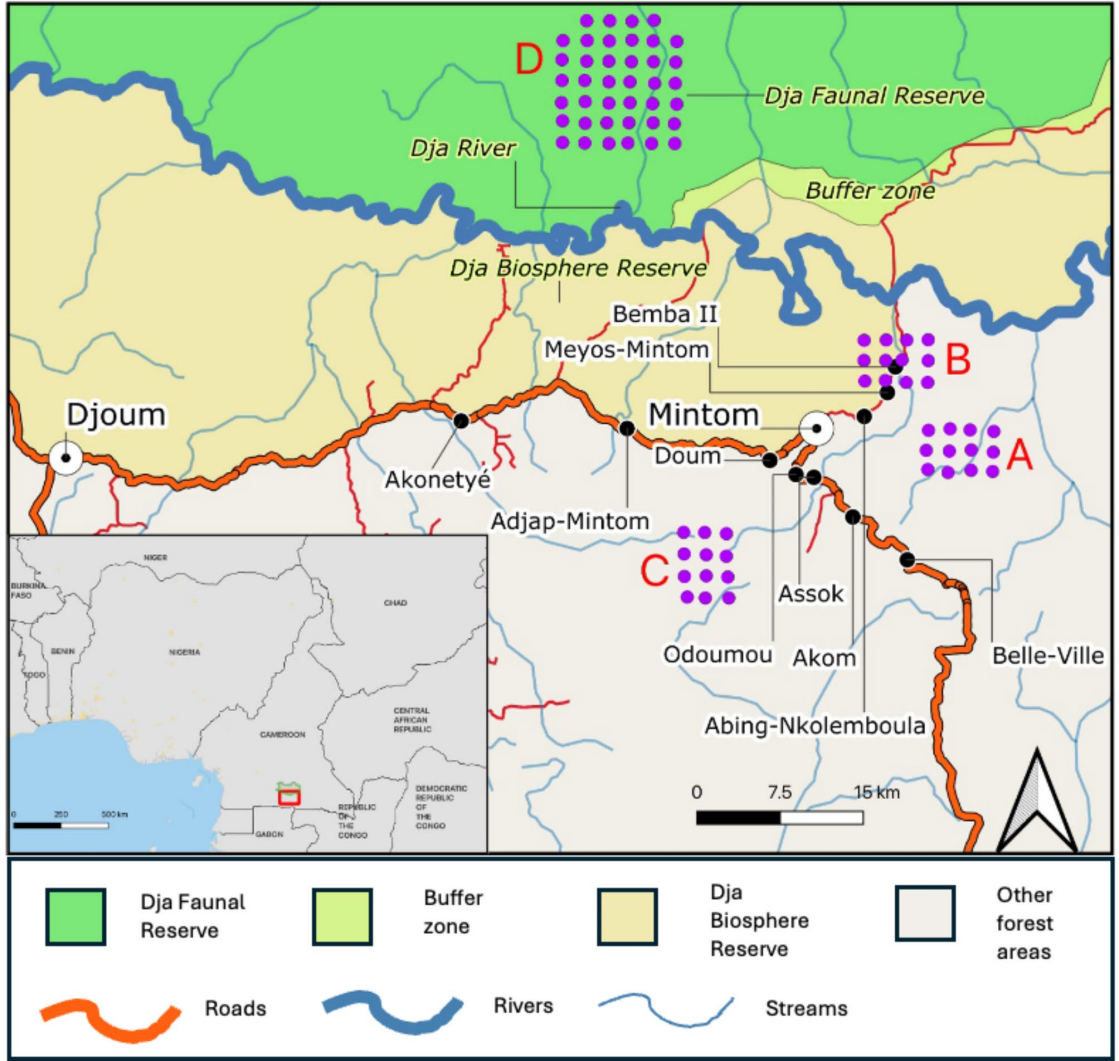
Study area, with the location of the three camera trap grids (A = East, B = Central, C = South) placed in Hunting Areas (HA) and grid (D) in the Non-Hunting Area (NHA) in Dja Faunal Reserve for comparison (Bruce et al.^20,21^). The map was created using QGIS version 3.16.0-Hannover (https://qgis.org/en/site/) from public domain map datasets from Open Street Map (www.openstreetmap.org), diva-gis (diva-gis.org) and Natural Earth (http://www.naturalearthdata.com) and the published map of the Dja Biosphere Reserve^22^.

This study was conducted in the region immediately south of the Dja River, in Southern Cameroon, north of the Gabon border (Fig. 1). The area is characterised by tropical vegetation comprising *terra firme* forests and monodominant stands of African zebrawood (*Gilbertiodendron dewevrei*). The topography consists of sloping terrain with gently rolling hills ranging from 250 to 800 m. The area has seasonally inundated areas, swamps and small rivers that flow into the Dja River.

The climate is equatorial, with four seasons: a long rainy season from August to November, a long dry season from November to March, a short rainy season from March to May, and a short dry season in June and July. Annual average rainfall in Djoum, a town approximately 83 km east of the study area, is between 1,500 and 2,000 mm. Average temperatures remain around 25 °C throughout the year^18^.

### Population and hunting territories

The study involved ten villages with an estimated total population of 736 individuals^19^. The nearest village was 17 km from the DFR, while the farthest village was 30 km away. The core hunting areas for all study villages were determined through participatory mapping conducted by the communities (see^18^). The maps, which included hunting trails, village hunting areas, and camps, were digitised using ArcMap 10.2. The accuracy of the maps was assessed by comparing them with the coordinates obtained using a handheld GPS receiver. The hunting territories are utilised throughout the year and consist of two main areas, namely the East and South, shared by all the villages^18^. Our previous community-based study showed that most of the 2,245 hunted carcasses recorded over 5 months were trapped (65.77% ± 16.63) compared to gun hunting (25.56% ± 17.72)^19^. The Baka hunting areas border large areas of unutilised forest, with the nearest permanent settlements more than 75 km to the east and south.

### Camera trap surveys

A camera trap survey was conducted from January to June 2018 in the hunting area (HA) using 36 Bushnell NatureView cameras (Fig. 1) equipped with low-glow infrared flash lighting to minimise the risk of startling animals. Single grids of 12 cameras were placed over the core hunting areas in the eastern and southern hunting areas. The cameras’ straight-line distances to the villages ranged between 5.1 and 12.5 km (East) and 7–14 km (South). Our household surveys^18,19^ indicated hunting is also undertaken around the villages by employing a sophisticated system of fences around small agricultural fields to funnel animals into snares. To examine the effect of more localised hunting around villages, a third grid (Central) of 12 cameras was placed surrounding one of the Baka villages (Bemba II) with a distance between 800 m and 3.7 km from the village. Grid placement within core hunting areas was determined using ArcMap 10.2 and downloaded to a handheld GPS device for positioning in the field. The cameras were spaced 2 km apart, positioned within 200 m of each grid point aimed at a game trail at a height of 30–45 cm above the ground and set to take three consecutive images per trigger with a two-second delay. Data from the three grids were combined to form the HA dataset.

The HA camera trap survey results were compared with data collected from the non-hunted areas (NHA) surveyed between May and June 2017 in the DFR^20^. The DFR is north of Baka hunting areas, with the Dja River forming a barrier between the HA and NHA. The 2017 survey deployed two camera trap grids: one in the northern sector of the DFR and one in the southern sector, with signs of hunting activity systematically recorded for both areas (see^20^). The southern sector grid was selected as the comparative area (NHA) for this study due to the very low levels of hunting activity recorded, its closer proximity (< 25 km) and similar habitat to HA. The NHA survey used 40 cameras spaced 2 km apart, positioned within 200 m of each grid point in an opening with sufficient field of view, and set at 30–45 cm above the ground. Camera settings for both HA and NHA were identical.

### Hunting data

Hunting data were collected concurrently with the camera trap survey (see^19^). In each of the 10 Baka villages, a village recorder (VR) visited participating hunters’ households at the end of each day to record the species hunted, the hunting methods used on any returned carcasses and whether the carcass was consumed or sold^19^.

### Data analysis

The captured images were sorted and identified using Camelot software^23^. Independent events, defined as occurring at least 60 min apart, were selected for analysis. Despite potential detection biases and sampling errors related to smaller mammals (< 0.5 kg), squirrels were included to compare differences between areas rather than estimate absolute abundance. We combined Lady Burton’s rope squirrel (*Funisciurus isabella*) and fire-footed squirrel (*Funisciurus pyrropus*) as ‘*striped squirrels*’ because they were difficult to differentiate in some camera trap images. Sampling effort for each grid was calculated and compared through rarefied species accumulation curves in the ‘*iNEXT*’ package^24^, and alpha diversity was measured using Simpson’s and Shannon-Weiner indices in the ‘*Vegan*’ package^25^ in R v4.1.1. Beta diversity between the HA and NHA was evaluated using Sørensen and Jaccard indices using the components ‘replacement’ and ‘richness difference’ obtained in the ‘*adespatial’* package^26^ in R.

For species detected in both HA and NHA, we examined the proportion of sites occupied and the influence of hunting (Hu) using hierarchical modelling that considers detection probability. Detection histories were truncated into 10-day sampling occasions for both sites, and we used a standard set of models for all species. Single-season occupancy models were fitted to the data using the package ‘*unmarked*’^27^ according to a two-stage process: (1) estimating detection probability (.*p*) evaluating a hunting model (Hu) and a null model, whilst assuming constant occupancy ($\psi$); (2) Applying the most supported detection model to estimate occupancy in each area ($\psi$). Model dispersion was calculated using the MacKenzie and Bailey^28^ goodness-of-fit test with 1,000 simulations for each model in r package ‘*AICcmodavg*’^29^. Over-dispersed models (c-hat > 2) were rejected. Models were ranked using the Akaike Information Criterion (AIC) in the package ‘*MuMin*’^30^, with models ΔAIC > 2 considered non-competing.

For unmarked species where individuals cannot be identified, relative abundance indices (RAIs, captures per trap effort) are widely used as a proxy for actual abundance in camera trap studies^31^. RAIs are controversial as they assume that detection probabilities are spatially and temporally constant across species. It has been demonstrated that significant detection variability is associated with camera traps and differences in RAIs, which are attributed to differences in abundance and can often be due to imperfect detection^32^. Applying n-mixture models to count data, incorporating detection probability, has significantly improved RAIs estimating relative abundance^33–36^. We derived relative abundance models from count data using a hierarchical approach similar to occupancy modelling. Optimal n-mixture models explaining detection probability were used to examine the influence of hunting on relative abundance and count distribution (P, NB, ZIP) for each species in the ‘*unmarked*’ package^27^.

The relative abundance of each species for each camera was calculated as a relative abundance index (RAI) per 100 trap days to enable the examination of the relationship between RAI and (1) distance from hunting villages and (2) village offtake. Regression analyses examined faunal depletion using the independent variable of “distance from village”, with camera grid as a random effect. The relationship between hunting offtake and abundance was examined through regression of village offtake and abundance (RAI) for the camera grid in the village hunting area. We only included offtake, which had been trapped, as we consider camera traps a more appropriate measure of availability for trapping rather than gun hunting, which also targets arboreal species.

### Permits and ethics

Permission to undertake field work was granted by the Cameroonian Ministry of Scientific Research and Innovation (MINRESI), via the nationally represented Center for International Forestry Research (CIFOR). Authorisation to work with human subjects was covered by Arrete No. 00034/A/MINATD/DAP/SDLP granted by the Ministère de L’Administration Territoriale et de La Decentralisation of the Government of Cameroon to ZyL. We followed the principle of free, prior and informed consent (FPIC) in which all hunters in our study freely participated in our project. We obtained informed consent from all participants who could stop contributing to the project. Participants were made aware that their participation would have no negative consequences. They were also informed that their identity would be kept anonymous and all personal information provided would be treated confidentially. Our study also followed the Social Research Association’s ethical research guidelines. Although children were present in the participatory workshops because these meetings were open to everyone in the villages (see below), no one under 18 (minors according to Cameroon law) was directly involved in our study.

## Results

### Camera trap survey

A total of 62,096 images were captured in HA grids, representing 6,532 independent events. These included at least 52 species, with 34 mammals (Supplementary Table S1). HA survey effort amounted to 3,856 trapping days, with an average of 107 days per camera, with each grid exceeding 1,000 days of camera trapping effort^37^. Most cameras (30 out of 36) were operational for > 85% of the survey duration; the remaining six malfunctioned. The comparative study in the DFR (NHA) had a survey effort of 3,371 camera trap days (mean 84 /days camera) with eight cameras excluded from further analysis due to malfunction^20^. In HA, rodents (*N* = 1,914, 33%), ungulates (*N* = 1,827, 32%) and primates (*N* = 1,623, 28%) accounted for 93% of the independent observations, with carnivores accounting for 6% (*N* = 347). Rainfall patterns across HA and NHA were generally consistent between the 2017 and 2018 survey years (Fig. S1).

### Within hunted areas

**Fig. 2:**
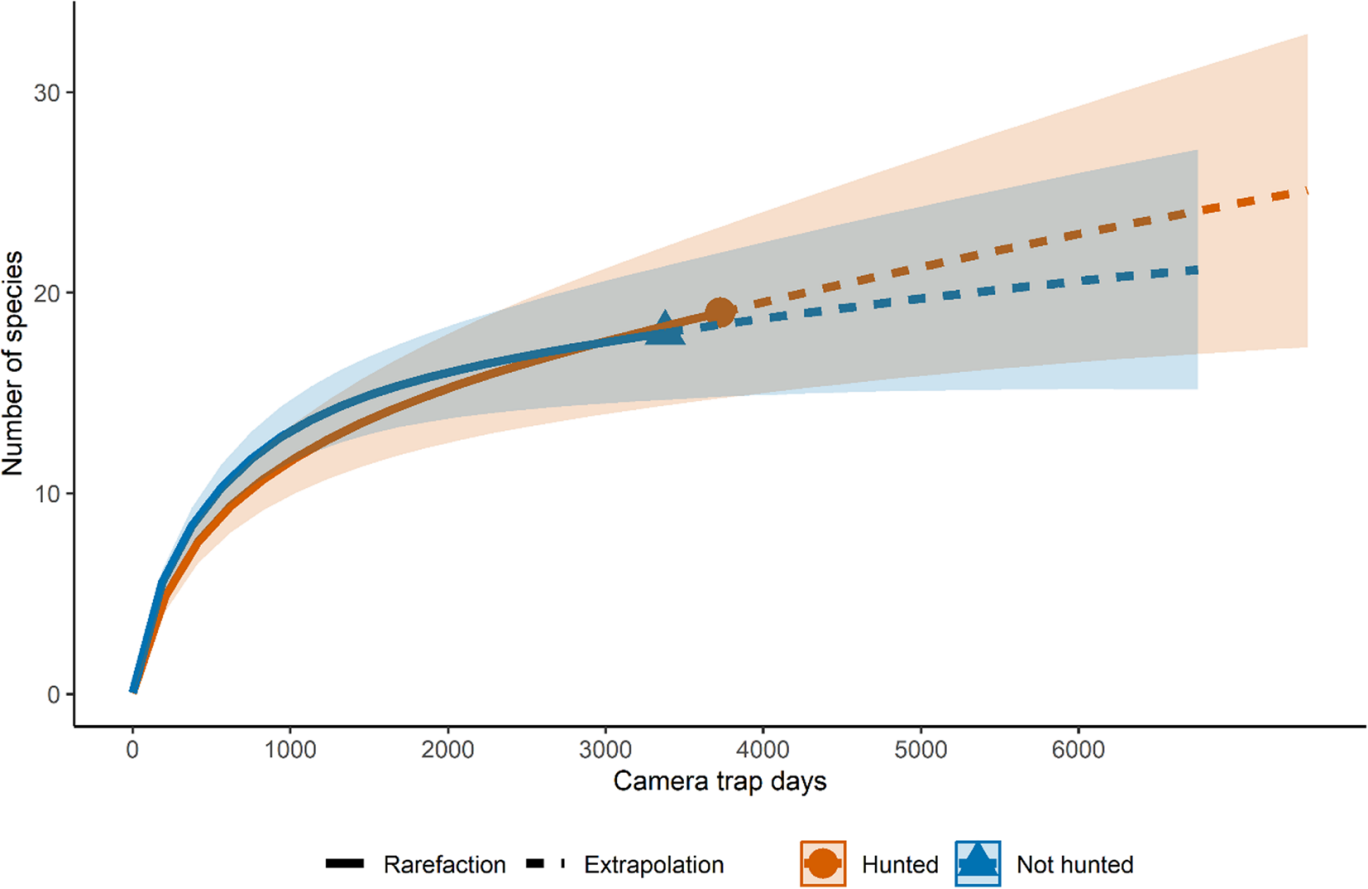
Rarefied species accumulation curve for terrestrial mammals in hunting and non-hunting areas. Confidence intervals are 95%, and curves are extrapolated (dashed lines) to approximately double the camera trap days.

Although all the HA grids had over 1,000 camera trap days, only the eastern grid reached asymptote in its species accumulation curve (Fig. 2). The central grid located over the villages exhibited the lowest alpha diversity indices (Simpson’s = 0.77, Shannon-Weiner = 1.79) compared to the other grids (Table 1). A weak but significant positive relationship was observed between alpha diversity indices and distance from the village (Simpsons, R^2^ = 0.10, F_(1, 28)_ = 4.36, *p* = 0.046; Shannon-Weiner, R^2^ = 0.24, F_(1, 28)_ = 10.29, *p* = 0.003).

**Table 1:** $\alpha$-diversity indices for individual hunting areas (East, Central, South), and combined hunting (HA) and non-hunting (NHA) areas, the latter located in the Dja Faunal Reserve.

	East	Central	South	HA	NHA
Simpson diversity	0.82	0.77	0.84	0.83	0.78
Shannon diversity	2.08	1.79	2.13	2.01	2.08

### Between hunted and non-hunted areas

Five species were detected in the HA but not in the NHA, while eight were recorded in the NHA but not in the HA (Table 2). The African golden cat (*Caracal aurata*), sitatunga (*Tragelaphus spekii*), and white-bellied duiker (*Cephalophus leucogaster*) were recorded as carcasses by the village recorders but were not detected by the camera traps in HA.

**Table 2:** Mammal species exclusively detected by camera trap surveys in either hunted or non-hunted areas in Southern Cameroon.

Hunted	Non-hunted
African civet	African forest elephant
Congo clawless otter	African golden cat
Galago spp	Black colobus
Honey badger	Bongo
Moustached guenon	Forest buffalo
	Sitatunga
	Western tree hyrax
	White-bellied duiker

The scientific names of the species are listed in Table S1.

Alpha diversity indices were similar for both areas, with HA having a higher Simpson’s index and NHA having a higher Shannon-Weiner index (Table 1). Species richness accounted for 65% of the difference in beta diversity between the HA and NHA, and replacement accounted for 35% (Table 3).

**Table 3:** $\beta$-diversity between hunted and non-hunted areas, showing the proportion of difference due to richness and replacement.

	Beta diversity	Replacement	Richness	Replacement/beta	Richness/beta
Jaccard	0.33	0.12	0.22	0.35	0.65
Sorenson	0.25	0.09	0.16	0.35	0.65

#### Occupancy

Detection probabilities differ considerably between species, with the lowest probability for Bates’ pygmy antelope (*Nesotragus batesi*, *p* = 0.01) and the highest for blue duiker (*Philantomba monticola*, *p* = 0.73) (Table 4). Hunting area was the explanatory variable of occupancy for all large primates, with mandrills (*Mandrillus sphinx*) showing substantially higher occupancy in HA ($\psi$ = 0.90, 95% CI 0.73–0.97) compared to NHA ($\psi$ = 0.03, 95% CI 0.01–0.18). Western lowland gorillas and chimpanzees (*Pan troglodytes*) had approximately half the estimated occupancy in HA compared to NHA. Large carnivores such as the African golden cat and leopard (*Panthera pardus*) had a higher occupancy in NHA than HA (Table 4). For those twenty-two species which constituted more than 90% of the village offtake (Table S2), only yellow-backed duiker (*Cephalophus silvicultor*), red river hog (*Potamochoerus porcus*) and water chevrotain (*Hyemoschus aquaticus*) demonstrated hunting as the explanatory variable for lower occupancy.

**Table 4:** Occupancy ($\psi$) and detection probabilities (*.p*) of mammal species in hunted (H) and non-hunted (NH) areas in Southern Cameroon.

Species	($\psi$)	*.p*	($\psi$) model	*p* model	$\psi$ C-hat	$\psi$ model* ΔAIC*	Competing $\psi$ model
African forest elephant							
H	[0]						
NH	0.63 (0.39–0.82)	0.15 (0.10–0.22)					
African brush-tailed porcupine	0.88 (0.77–0.94)	0.46 (0.42–0.49)	NULL	NULL		0.60	Hu
Striped and sun squirrels							
H	0.93 (0.77–0.98)	0.47 (0.41–0.52)	Hu	Hu	1.25	51.4	
NH	0.06 (0.01–0.20)	0.01 (0.01–0.02)					
African giant squirrel			Hu	Hu	2.11	4.09	
H	0.60 (0.42–0.76)	0.35 (0.29–0.42)					
NH	0.03 (0.01–0.18)	0.01 (0.01–0.03)					
Emin’s pouched rat		0.50 (0.46–0.54)	Hu	NULL	1.21	0.87	NULL
H	0.96 (0.80–0.99)						
NH	0.80 (0.64–0.90)						
Mandrill		0.34 (0.29–0.40)	Hu	NULL	1.54	2.99	
H	0.9 (0.73–0.97)						
NH	0.03 (0.01–0.18)						
Western lowland gorilla			Hu	Hu	0.62	2.26	
H	0.43 (0.09–0.85)	0.22 (0.15–0.31)					
NH	0.85 (0.01–0.99)	0.12 (0.08–0.18)					
Central chimpanzee			Hu	Hu	1.75	2.15	
H	0.55 (0.37–0.72)	0.19 (0.13–0.26)					
NH	0.84 (0.65–0.94)	0.33 (0.03–0.27)					
White-bellied pangolin	0.41 (0.29–0.55)	0.18 (0.13–0.24)	NULL	NULL	–	–	–
Giant pangolin	0.62 (0.31–0.85)		NULL	Hu		0.60	Hu
H		0.04 (0.02–0.09)					
NH		0.09 (0.05–0.17)					
African palm civet	0.60 (0.42–0.75)	0.14 (0.10–0.18)	NULL	NULL		1.16	Ha
Leopard			Hu	Hu	0.54	9.22	
H	0.05 (0.01–0.31)	0.01 (0.01–0.04)					
NH	0.57 (0.19–0.88)	0.08 (0.04–0.17)					
African golden cat							
H	[0]						
NH	0.46 (0.22–0.73)	0.11 (0.06–0.20)					
Servaline genet	0.75 (0.62–0.86)	0.29 (0.18–0.26)	NULL	NULL		0.80	Ha
Black-footed mongoose			Hu	Hu	1.84	3.26	
H	0.48 (0.31–0.66)	0.17 (0.12–0.25)					
NH	0.88 (0.67–0.96)	0.29 (0.24–0.34)					
Marsh mongoose	0.19 (0.11 – 0.32)	0.19 (0.12–0.27)	NULL	NULL	–	–	–
Long-nosed mongoose	0.31 (0.21–0.44)	0.23 (0.18–0.30)	NULL	Ha	–	–	–
Cameroon cusimanse	0.45 (0.31–0.59)		NULL	Hu		0.54	Ha
H		0.22 (0.15–0.31)					
NH		0.12 (0.08–0.18)					
Red river hog			Hu	Hu	2.09	2.83	
H	0.17 (0.07–0.35)	0.02 (0.01–0.04)					
NH	0.90 (0.69–0.97)	0.29 (0.24–0.34)					
Forest buffalo							
H	[0]						
NH	0.03 (0.01–0.19)	0.23 (0.04–0.65)					
Bongo							
H	[0]						
NH	[0.06]	0.006 (0.001–0.026)					
Sitatunga							
H	[0]						
NH	0.08 (0.02–0.31)	0.13 (0.02–0.47)					
Bate’s pygmy antelope			Hu	Hu	0.14	7.93	
H	0.09 (0.01–0.51)	0.01 (0.01–0.03)					
NH	0.80 (0.01–0.99)	0.04 (0.01–0.15)					
Blue duiker	1 (0)	0.73	NULL	NULL	–	–	–
Peter’s duiker	0.95 (0.87–0.99)	0.61 (0.57–0.64)	NULL	NULL	–	–	–
Bay duiker	0.86 (0.74–0.92)	0.38 (0.35–0.42)	NULL	NULL		2.13	
White-bellied duiker							
H	[0]						
NH	0.72 (0.53–0.85)	0.28 (0.23–0.34)					
Black-fronted duiker	0.20 (0.11–0.33)	0.16 (0.10–0.25)	NULL	NULL	–	–	–
Yellow-backed duiker			Hu	Hu	1.82	3.39	
H	0.52 (0.33–0.69)	0.08 (0.05–0.12)					
NH	0.90 (0.66–0.98)	0.33 (0.28–0.039)					
Water chevrotain			Hu	Hu	0.99	3.20	
H	0.07 (0.02–0.23)	0.26 (0.11–0.49)					
NH	0.40 (0.25–0.57)	0.43 (0.35–0.51)					

[0] denotes species was not detected either in H = hunted; NH = non-hunted, occupancy and detection probabilities modelled for the area where species was detected. The scientific names of the species are listed in Table S1.

#### Relative abundance models

The negative binomial distribution was selected for all species with a probability of detection greater than 0.01. However, detection probabilities for relative abundance were generally lower than for the single-season occupancy models. Five species with detection probabilities below 0.01 were excluded from further analysis (Table 5). For the remaining species, confidence intervals were generally large. However, the intention was to understand the differences and explanatory variables of relative abundance rather than determine abundance itself.

**Table 5:** N-mixture relative abundance models and detection probabilities of mammal species in hunted (H) and non-hunted (N) areas in Southern Cameroon.

Species	Relative abundance	Relative abundance model	*.p*	*p* model	Count distribution	RAM c-hat	*ΔAIC* RAM	Competing model
African brush-tailed porcupine		Hu	0.04 (0.03–0.05)	NULL	NB	1.97	2.53	
H	37.61 (24.12–58.61)							
NH	20.00 (13.04–30.68)							
Marsh mongoose		Hu	0.08 (0.02–0.04)	NULL	NB	1.17	0	NULL
H	11.07 (1.99–61.53)							
NH	1.09 (0.15–8.2)							
Black-footed mongoose		Hu		Hu	NB	1.48	1.5	NULL
H	12.66 (5.33–30.04)		0.02 (0.01–0.04)					
NH	29.92 (12.98–68.94)		0.03 (0.02–0.05)					
Peter’s duiker		Hu		Hu	NB	1.98	44.18	
H	4.98 (3.38–7.36)		0.02 (0.02–0.32)					
NH	45.94 (33.36–63.14)		0.15 (0.14–0.16)					
Bay duiker	24.5 (16.03–37.35)	NULL	0.04 (0.02–0.04)	NULL	NB		1.9	Hu
Yellow-backed duiker		Hu		Hu	NB	1.39	26.37	
H	4.06 (1.99–8.27)		0.01 (0.01–0.05)					
NH	27.69 (15.69–48.58)		0.02 (0.01–0.03)					
Black-fronted duiker	4.33 (0.75–24.93)	NULL	0.01 (0.01–0.05)	NULL	NB		1.94	Hu
Emin’s pouched rat		Hu	0.07 (0.06–0.09)	NULL	NB	2.01	6.22	
H	34.51 (22.27–53.46)							
NH	10.15 (6.66–15.48)							
Cameroon cusimanse		Hu	0.07 (0.06–0.09)	NULL	NB	1.63	0.5	NULL
H	17.06 (7.74–41.54)							
NH	7.12 (2.75–18.49)							
Servaline genet	24.3 (5.86–101.06)	NULL	0.08 (0.02–0.3)	NULL	NB		2.27	
Western lowland gorilla		NULL	< 0.01					
Long-nosed mongoose	8.96 (3.46–23.16)	NULL	0.01 (0.006–0.03)	NULL	NB		0.85	Hu
Water chevrotain		Hu	0.03 (0.02–0.05)	NULL	NB	1.46	5.46	
H	0.53 (0.19–14.50)							
NH	14.99 (5.27–42.63)							
Mandrill		Hu	0.05 (0.04–0.05)	NULL	NB	2.01*	81.65	
H	67.40 (48.02–94.60)							
NH	0.08 (0.01–0.59)							
African palm civet	18.9 (4.67–76.61)	NULL	0.05 (0.01–0.20)	NULL	NB			
Central chimpanzee	21.7 (12.70–33.87)	NULL	0.02 (0.01–0.03)	NULL	NB		0.76	Hu
White-bellied pangolin			< 0.01					
Blue duiker	44.8 (35.79–56.15)	NULL	0.08 (0.07–0.09)	NULL	NB		1.71	Hu
Red river hog		Hu		Hu	NB	1.47	38.63	
H	0.56 (0.20–1.59)		0.002 (0.001–0.004)					
NH	17.12 (9.67–30.31)		0.03 (0.01–0.03)					
Giant pangolin			< 0.01					
Striped and sun squirrels		Hu		Hu	NB	1.69	60.23	
H	28.68 (18.09–45.47)		0.04 (0.03–0.05)					
NH	0.25 (0.14–0.82)		0.004 (0.001–0.02)					
African giant squirrel		Hu		Hu	NB	1.37	23.79	
H	14.24 (6.20–32.76)		0.03 (0.02–0.05)					
NH	0.21 (0.04–1.02)		0.004 (0.001–0.01)					
Leopard								
H	0.44 (0.04–4.55)	Hu	0.007 (0.004–0.02)	Hu	NB	1.95	10.12	
NH	12.70 (3.63–44.44)		0.01 (0.001–0.02)					
Bate’s pygmy antelope			< 0.01					
African golden cat								
H	[0]							
NH	0.69 (0.02–19.76)		0.04 (0.001–0.58)					
African forest elephant								
H	[0]							
NH	7.37 (2.84–19.08)		0.01 (0.006–0.03)					
Forest buffalo								
H	[0]							
NH	0.02 (0.002–0.119)		0.23 (0.05–0.65)					
White-bellied duiker								
H	[0]							
NH	9.93 (4.34–22.76)		0.02 (0.01–0.04)					
Sitatunga								
H	[0]							
NH	0.08 (0.02–0.41)		0.01 (0.02–0.48)					
Bongo			< 0.01					

Goodness of fit (c-hat) and *ΔAIC* given for relative abundance models. Where c-hat > 2 NULL model accepted and c-hat and *ΔAIC* are marked as ‘–’. H = hunted; NH = non-hunted. The scientific names of the species are listed in Table S1.

**Fig. 3:**
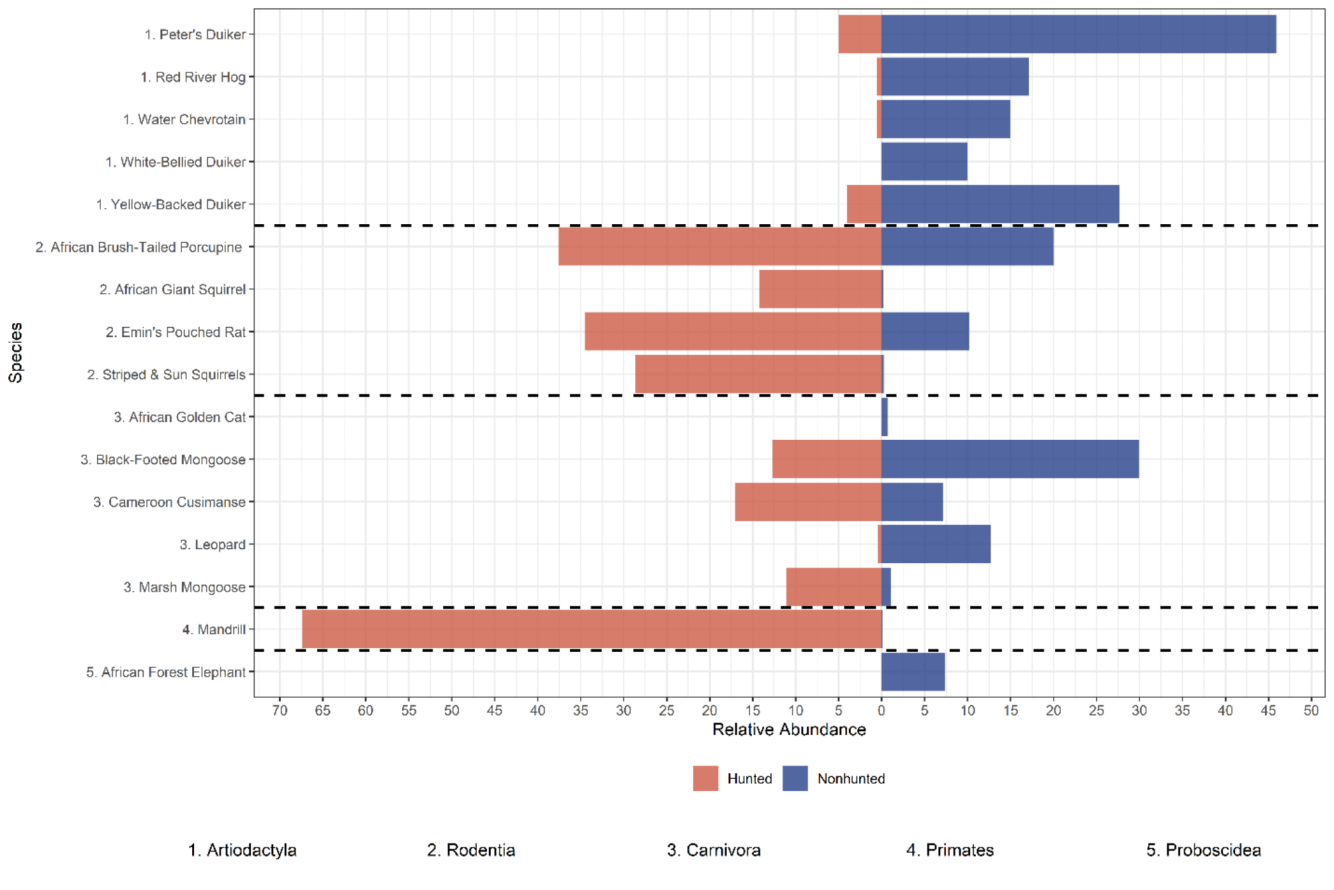
N-mixture relative abundance models for species with hunting area as the explanatory variable for relative abundance.

N-mixture models showed that hunting impacted the relative abundance of 13 species present in both areas with a probability of detection greater than 0.01; in seven species, hunting had a positive effect on abundance, and in six species, hunting had a negative effect (Fig. 3).

#### Carnivores

Leopards (*Panthera pardus*) showed higher relative abundance in NHA than HA, although estimated detection probabilities in HA were very low. Mesopredators showed higher abundance in HA for marsh mongoose (*Atilax paludinosus*), Cameroon cusimanse (*Crossarchus platycephalus*), and long-nosed mongoose (*Herpestes naso*). In comparison, black-footed mongoose (*Bdeogale nigripes*) had significantly higher abundance in NHA. Servaline genets (*Genetta servalina*) and African palm civets (*Nandinia binotata*) showed no differences in abundance between areas.

#### Rodents

Rodents displayed higher relative abundance in HA, with hunting as the explanatory variable across all species. Emin’s pouched rat (*Cricetomys emini*) had approximately three times higher relative abundance in hunting areas, while African brush-tailed porcupines (*Atherurus africanus*) had almost doubled. Red-legged sun squirrels (*Heliosciurus rufobrachium*), striped squirrels, and African giant squirrels (*Protoxerus stangeri*) had two orders of magnitude higher relative abundances in HA than NHA.

#### Ungulates

Significant differences in relative abundance were observed in ungulate species. Peter’s duiker (*Cephalophus callipygus*), yellow-backed duiker (*Cephalophus silvicultor*) and water chevrotain (*Hyemoschus aquaticus*) all showed lower abundance in HA compared to NHA. Peter’s duiker had an order of magnitude higher predicted relative abundance in NHA than HA. Red river hogs (*Potamochoerus porcus*) were also significantly less abundant in HA than NHA. Blue and bay duikers (*Cephalophus dorsalis*) showed no significant difference between areas, and a null model was selected for both.

#### Large primates

Only mandrills had hunting as the explanatory variable for relative abundance among large primates. Western lowland gorillas had a detection probability of less than 0.01, and central chimpanzees had a null model for relative abundance.

#### Relative abundance indices

We found no significant relationship between RAI and distance from the village for any species with more than two independent detections, with the camera grid not having an effect. There was a significant positive relationship between RAI and hunting frequency with snares (R^2^ = 0.69, F(1, 32) = 74.59, *p* ≤  0.001) (Fig. S2).

## Discussion

Our study revealed that species richness was similar between areas experiencing minimal hunting pressure and those hunted for decades by sedentarised Baka Pygmies in southeastern Cameroon. However, despite comparable species richness, there were significant differences in species occupancy and relative abundance across several species groups. Hunting areas showed a marked decrease in large carnivores and some ungulate species, with a significant increase in rodents and meso-predators compared to the non-hunted area. Our previous community-based study showed that most of the 2,245 recorded animals hunted by the Baka communities were ungulates, with trapping being the predominant hunting method^19^. Approximately 67% of the carcasses were consumed partially or entirely by the Baka households. The estimated annual wild meat extraction by the communities was around 50,000 kg from a total hunting area of 2,052 km^2^^18^. There is significant variation in mammalian biomass in tropical forests, but taking a lower estimate of 998 kg/km^2^ of available biomass^31^, the annual offtake by the Baka communities represents approximately 2.4% of the potentially available biomass from the defined hunting areas^32^.

The spatial context of hunting and relatively low offtake compared to other studies may be the reason we found no strong evidence for village-centred gradients of defaunation, with a very weak effect of distance to the village on diversity indices and no effect on relative abundance for any species^19,33^. Diversity indices between hunted and non-hunted areas were comparable and indicated a limited impact of hunting on species richness. Indeed, species particularly vulnerable to hunting, such as the giant pangolin (*Smutsia gigantea*), had relatively high occupancy in the hunting areas^34^. Localised harvests and apparent maintenance of species richness in these long-term hunted areas are likely to be a function of the spatial context of the hunting areas and heterogeneity in hunting pressure across the region^9,10,16,35,36^. All hunting areas utilised by the Baka border large areas of unutilised forest, with the nearest permanent settlements more than 75 km to the east and south. Even within the relatively small hunting areas, hunting is undertaken from defined trails, with most areas not accessible to hunting^18^. Therefore, these large adjacent areas likely act as sources of biodiversity and maintain species richness and occupancy as a function of source-sink dynamics.

Whilst the spatial context of hunting and source populations seems to be maintaining overall species richness and offtake, our results indicate that there may be pronounced changes in wildlife assemblages at all trophic levels in the Baka hunting areas. Sustained hunting produces shifts in species composition in many tropical forest systems and can significantly affect forest structure, regeneration and biogeochemical cycles^12,15,38,39^. However, these effects were documented in areas with higher hunting pressure than our study and seemingly without source-sink dynamics. Hunting often leads to changes in the available prey base, mainly through the decline of medium-bodied duikers, negatively impacting large carnivore numbers^40^. Indeed, leopards had a much lower occupancy in hunting areas than those without hunting. Our camera traps did not detect the African golden cat, but the community-based reporting system reported four carcasses^19^, representing significant offtake and a likely negative impact of hunting on the species^21^. Declines in apex predators can result in ecological niche adjustments, where mesopredators increase abundance^12^. The black-legged mongoose is considered sensitive to disturbance^20^ and decreased in hunting areas. Other mongooses and the Cameroon cusimanse displayed significantly higher occupancy and abundance in hunting areas, suggesting ecological release. Genets and civets showed no difference between hunted and non-hunted areas.

The apparent absence of some large ‘forest architects,’ such as elephants and forest buffalos, in the hunting areas cannot be solely attributed to subsistence hunting. During the study, only one elephant skeleton was found in the hunting areas, and there was no evidence of recent elephant signs. Forest buffalos were recorded at very low abundance in areas without hunting, and their absence in the hunting areas may be due to the lack of suitable habitat, notably clearings and open forest stands^41^. The decline of elephants in the region, which is linked to a significant increase in commercial poaching, has probably contributed to their absence in the hunting areas^42^, but undetected masting events in NHA could also contribute to the differences observed in this study. The difference in abundance of mandrills between the two areas is unlikely due to hunting, given that the Dja River is at the limit of the known species range^43^. However, the high occupancy and relative abundance of mandrills in the hunting areas suggest that hunting minimally affects it. Mandrills were hunted more frequently than red river hogs, with 39 carcasses recorded by the village recorders across the 10 villages (Supplementary Table S2).

The most significant differences between hunted and non-hunted areas were observed for medium-bodied frugivores and small seed predators. Changes in occupancy and abundance in this middle trophic guild can have cascading effects on higher and lower trophic guilds (due to ecological release) and even forest structure and regeneration^13^. The reduction in seed dispersal by large and medium-sized frugivores can impact the forest’s regeneration; approximately 85% of trees in African tropical forests depend on animals for dispersal^44^. The yellow-backed duiker, water chevrotain, and red river hog showed significant declines in occupancy and abundance in hunting areas. The diurnal Peter’s duiker, which has a more critical seed dispersal role than the sympatric nocturnal bay duiker^45^, exhibited a much lower relative abundance in hunted areas compared to areas without hunting. Changes in the middle trophic layer have cascaded to lower layers with significant increases in hunting areas in the occupancy and particularly the abundance of granivores. Pouched rats, porcupines, and squirrels are all present at much higher levels in hunting areas than in non-hunting areas, with presumably significantly higher levels of seed predation in the hunted area than in the non-hunted area^14^.

Quantifying the effect of wild meat hunting on tropical forest fauna is difficult, information is often lacking on species abundance, and variation in mammalian biomass between areas means that extrapolating estimates between differing areas is often unsound^46,47^. Although recent methodological developments potentially offer means to derive accurate abundance estimates from unmarked forest animals using camera traps^48–50^, several studies have used a counterfactual approach similar to the one adopted in this study, where faunal richness and relative abundance of a hunted area is compared to that of an area which is subject to low levels or no hunting^13,14,51–55^. We used camera traps to estimate species richness and relative abundance between a hunted and a non-hunted area, surveyed one year apart. Ideally, these surveys would have been conducted concurrently, but the project on the hunting communities did not commence until after the DFR survey was completed. Nonetheless, we argue that our results indicate hunting-mediated differences between the two areas; the two areas are near each other (< 25 km), very similar in habitat, with rainfall patterns consistent between the two survey years. Both HA & NHA have areas of monodominant *Gilbertiodendron dewevrei*, which exhibits synchronous mast seed production every 2–3 years^56^. These mast fruiting events provide a temporally abundant food source for many large mammals and have a pronounced effect on the spatial and temporal patterns of resource use, particularly on those species with large-ranging patterns, such as elephants and great apes^57^. No mast events occurred during the survey in HA. However, the presence or absence of masting events was not recorded during the NHA survey. Undocumented masting events in NHA are predicted to increase the abundance of elephants, chimpanzees, gorillas, and possibly red river hogs in the non-hunted areas^58^. Whilst masting events have been demonstrated in other forest systems to increase rodent abundance, we documented a significantly higher abundance of rodents in HA, which is the opposite of what would be predicted if there was a masting event in NHA during the survey period^59^. Species with smaller home ranges and fixed territories are unlikely to be affected in their spatial and temporal habitat use relative to masting events^20^. The results of this study should be viewed in the context of a single temporal survey event, and future studies should encompass multi-year and multi-season surveys.

This study indicates that low to moderate offtake seems to impact functional diversity even in systems where the spatial context of hunting maintains offtake and biodiversity. The short period over which this study was conducted means that the potential implications for forest structure and regeneration are impossible to assess, and long-term research is needed to accurately assess the impacts of hunting and its ramifications on forest structure and the provision of dietary protein for Indigenous Peoples^15,60^. The spatial management of hunting is easier to implement than a quota-based system and is likely more effective. However, this study shows that even when hunting is managed through established no-take zones, this may still have long-term implications for functional diversity. Protecting functional diversity and considering the ecological roles of species is crucial for sustainable hunting practices and preserving biodiversity in Indigenous territories. Collaboration between Indigenous communities, researchers, and policymakers is essential for addressing the complexities and ensuring sustainable wildlife management in these territories.

## Electronic supplementary material

Below is the link to the electronic supplementary material.

Supplementary Material 1

## Data Availability

The datasets used and/or analysed during the current study available from the corresponding author on reasonable request.
