# Supplementary material for: Subsistence hunting impacts wildlife assemblages and functional ecology in tropical forests
Source: Sci Rep. 2025 Jan 24;15:3091. doi: 10.1038/s41598-025-87162-w (PMC11760533; doi:10.1038/s41598-025-87162-w)
Supplement: Supplementary file 1 — Supplementary Material 1 [file 41598_2025_87162_MOESM1_ESM.docx]

**
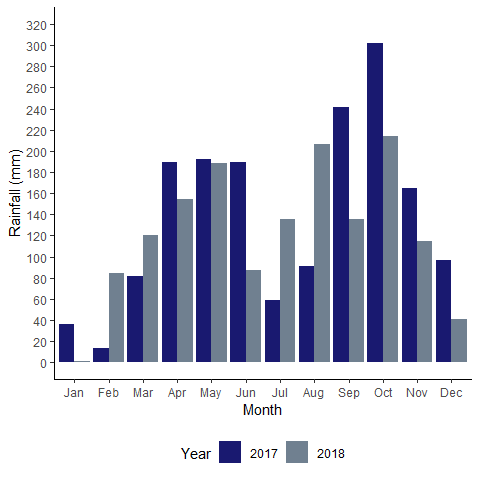
**

**Figure S1** Mean monthly rainfall for hunting areas and non-hunting areas in 2017 & 2018 (source. NASA/GLDAS/V021/NOAH/G025/T3H).

**Reference**

Rodell, M., P.R. Houser, U. Jambor, J. Gottschalck, K. Mitchell, C.-J. Meng, K. Arsenault, B. Cosgrove, J. Radakovich, M. Bosilovich, J.K. Entin, J.P. Walker, D. Lohmann, and D. Toll, The Global Land Data Assimilation System, Bull. Amer. Meteor. Soc., 85(3), 381-394, 2004. http://www.jstor.org/stable/26216951

**Figure S2** Percentage of village offtake by snaring relative to species’ RAI in village hunting area (R2 = 0.69, F (1, 32) = 74.59, p = <0.001).


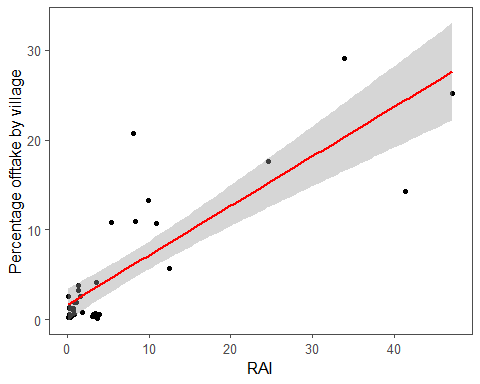


**Table S1** Species recorded in Hunting areas, number of working camera traps that detected the species, number of images and number of independent events.

| **Order** | **Species** | **No. of Traps (/30)** | **No. of Photos** | **Independent Observations** |
| --- | --- | --- | --- | --- |
| Bucerotiformes | White-crested hornbill (*Horizocerus albocristatus*) | 2 | 6 | 2 |
| Carnivora | African civet (*Civettictis civetta*) | 2 | 12 | 3 |
| Carnivora | African palm civet (*Nandinia binotata*) | 14 | 179 | 41 |
| Carnivora | Black-footed mongoose (*Bdeogale nigripes*) | 14 | 261 | 56 |
| Carnivora | Cameroon cusimanse (*Crossarchus platycephalus*) | 10 | 294 | 69 |
| Carnivora | Central African oyan (*Poiana richardsonii*) | 1 | 6 | 1 |
| Carnivora | Congo clawless otter (*Aonyx congicus*) | 1 | 3 | 1 |
| Carnivora | Honey badger (*Mellivora capensis*) | 2 | 24 | 3 |
| Carnivora | Leopard (*Panthera pardus*) | 1 | 6 | 1 |
| Carnivora | Long-nosed mongoose (*Herpestes naso*) | 13 | 369 | 62 |
| Carnivora | Marsh mongoose (*Atilax paludinosus*) | 8 | 149 | 34 |
| Carnivora | Servaline genet (*Genetta servalina*) | 21 | 281 | 76 |
| Cetartiodactyla | Bates's pygmy antelope (*Nesotragus batesi*) | 1 | 3 | 1 |
| Cetartiodactyla | Bay duiker (*Cephalophus dorsalis*) | 26 | 2,373 | 263 |
| Cetartiodactyla | Black-fronted duiker (*Cephalophus nigrifrons*) | 6 | 129 | 13 |
| Cetartiodactyla | Blue duiker (*Philantomba monticola*) | 30 | 8,665 | 1,243 |
| Cetartiodactyla | Peters's duiker (Cephalophus callipygus) | 27 | 2,390 | 266 |
| Cetartiodactyla | Red river hog (*Potamochoerus porcus*) | 5 | 48 | 6 |
| Cetartiodactyla | Water chevrotain (*Hyemoschus aquaticus*) | 2 | 225 | 13 |
| Cetartiodactyla | Yellow-backed duiker (*Cephalophus silvicultor*) | 15 | 334 | 28 |
| Columbiformes | Blue-headed wood dove (*Turtur brehmeri*) | 16 | 261 | 55 |
| Columbiformes | Lemon dove (*Aplopelia larvata*) | 1 | 6 | 2 |
| Galliformes | Black guineafowl (*Agelastes niger*) | 16 | 860 | 192 |
| Galliformes | Lathams's francolin (*Peliperdix lathami*) | 16 | 467 | 104 |
| Galliformes | Plumed guineafowl (*Guttera plumifera*) | 16 | 675 | 129 |
| Gruiformes | Nkulengu rail (*Himantornis haematopus*) | 10 | 227 | 35 |
| Passeriformes | Brown-chested alethe (*Chamaetylas poliocephala*) | 1 | 6 | 2 |
| Passeriformes | Green-breasted pitta (*Pitta reichenowi*) | 1 | 6 | 1 |
| Passeriformes | Grey-necked rockfowl (*Picatharthes oreas*) | 2 | 15 | 2 |
| Passeriformes | Orange-breasted forest robin (*Stiphrornis erythrothorax*) | 2 | 9 | 3 |
| Passeriformes | Red-tailed bristlebill (*Bleda syndactylus*) | 2 | 12 | 3 |
| Passeriformes | Yellow-lored bristlebill (*Bleda notatus*) | 2 | 12 | 3 |
| Passeriformes | Yellow-whiskered greenbul (*Eurillas latirostris*) | 2 | 6 | 2 |
| Pholidota | Giant pangolin (*Smutsia gigantea*) | 9 | 57 | 12 |
| Pholidota | White-bellied pangolin (*Phataginus tricuspis*) | 10 | 270 | 47 |
| Primates | Agile mangabey (*Cercocebus agilis*) | 9 | 1,731 | 291 |
| Primates | Central chimpanzee (*Pan troglodytes*) | 16 | 436 | 110 |
| Primates | Galago sp.c | 5 | 18 | 6 |
| Primates | Greater spot-nosed monkey (*Cercopithecus nictitans*) | 7 | 27 | 7 |
| Primates | Mandrill (*Mandrillus sphinx*) | 27 | 4,921 | 1,187 |
| Primates | Moustached guenon (*Cercopithecus cephus*) | 5 | 42 | 11 |
| Primates | Western lowland gorilla (*Gorilla gorilla*) | 5 | 39 | 11 |
| Rodentia | African brush-tailed porcupine (*Atherus africanus*) | 28 | 2,671 | 540 |
| Rodentia | African giant squirrel (*Protoxerus stangeri*) | 18 | 626 | 157 |
| Rodentia | Emin's pouched rat (*Cricetomys emini*) | 29 | 4,533 | 818 |
| Rodentia | Fire-footed rope squirrel (*Funisciurus pyrropus*) | 17 | 377 | 74 |
| Rodentia | Lady Burton's rope squirrel (*Funisciurus isabella*) | 22 | 962 | 217 |
| Rodentia | Red-legged sun squirrel (*Heliosciurus rufobrachium*) | 15 | 555 | 108 |
| Rodentia | Small rodent (Small rodent) | 17 | 704 | 208 |
| Squamata | Nile monitor (*Varanus niloticus*) | 1 | 3 | 1 |
| Testudine | Forest hinge-back tortoise (*Kinixys erosa*) | 1 | 3 | 1 |

**Table S2.** Hunted carcasses by species, number of carcasses and percentage of total returned carcasses recorded by village reporters from 10 Baka villages in south-eastern Cameroon over a 5-month period (Martin et al., 2020).

| Rank | Species | Number of carcasses | Percentage |
| --- | --- | --- | --- |
| 1 | **Blue duiker** | **473** | **20.93** |
| 2 | **Brush-tailed porcupine** | **333** | **14.73** |
| 3 | **Emin's pouched rat** | **220** | **9.73** |
| 4 | **Bay duiker** | **212** | **9.38** |
| 5 | **Peters's duiker** | **145** | **6.42** |
| 6 | **Tree pangolin** | **82** | **3.63** |
| 7 | **Forest hinge-back tortoise** | **72** | **3.19** |
| 8 | **Greater spot-nosed monkey** | **68** | **3.01** |
| 9 | **African palm civet** | **62** | **2.74** |
| 10 | **Moustached guenon** | **51** | **2.26** |
| 11 | **Duiker unknown** | **50** | **2.21** |
| 12 | **Mandrill** | **39** | **1.73** |
| 13 | **Red river hog** | **35** | **1.55** |
| 14 | **Gaboon viper** | **34** | **1.50** |
| 15 | **Monkey unknown** | **29** | **1.28** |
| 16 | **Ornate monitor** | **29** | **1.28** |
| 17 | **Long-nosed mongoose** | **27** | **1.19** |
| 18 | **Yellow-backed duiker** | **25** | **1.11** |
| 19 | **Mongoose unknown** | **23** | **1.02** |
| 20 | **Black-fronted duiker** | **22** | **0.97** |
| 21 | **Water chevrotain** | **21** | **0.93** |
| 22 | **Grey-cheeked mangabey** | **16** | **0.71** |
| 23 | **Sitatunga** | **15** | **0.66** |
| 24 | **Dwarf crocodile** | **14** | **0.62** |
| 25 | **Black Guineafowl** | **11** | **0.49** |
| 26 | **Black-footed mongoose** | **11** | **0.49** |
| 27 | **Black-necked spitting cobra** | **11** | **0.49** |
| 28 | **Bird unknown** | **11** | **0.49** |
| 29 | **African civet** | **10** | **0.44** |
| 30 | **Servaline genet** | **10** | **0.44** |
| 31 | **Mantled guereza** | **9** | **0.40** |
| 32 | **Crested mona monkey** | **8** | **0.35** |
| 33 | **Latham's Francolin** | **8** | **0.35** |
| 34 | **Agile mangabey** | **7** | **0.31** |
| 35 | **Congo clawless otter** | **7** | **0.31** |
| 36 | **Giant pangolin** | **6** | **0.27** |
| 37 | **Crested guineafowl** | **5** | **0.22** |
| 38 | **Kusimanse** | **5** | **0.22** |
| 39 | **Marsh mongoose** | **5** | **0.22** |
| 40 | **African golden cat** | **4** | **0.18** |
| 41 | **Chimpanzee** | **4** | **0.18** |
| 42 | **Spotted fowl unknown** | **4** | **0.18** |
| 43 | **White-bellied duiker** | **4** | **0.18** |
| 44 | **Bates's pygmy antelope** | **3** | **0.13** |
| 45 | **Black-casqued hornbill** | **3** | **0.13** |
| 46 | **Jameson's mamba** | **3** | **0.13** |
| 47 | **Western tree hyrax** | **3** | **0.13** |
| 48 | **Rodent unknown** | **2** | **0.09** |
| 49 | **Snake unknown** | **2** | **0.09** |
| 50 | **Squirrel unknown** | **2** | **0.09** |
| 51 | **Brown-cheeked hornbill** | **1** | **0.04** |
| 52 | **De Brazza's monkey** | **1** | **0.04** |
| 53 | **Unknown** | **1** | **0.04** |
| 54 | **Fish unknown** | **1** | **0.04** |
| 55 | **West African Potto** | **1** | **0.04** |
